# Safety and prescribing recommendations for verapamil in newly diagnosed pediatric type 1 diabetes (T1D): The CLVer experience

**DOI:** 10.1016/j.jcte.2024.100352

**Published:** 2024-05-18

**Authors:** Laya Ekhlaspour, Bruce Buckingham, Colleen Bauza, Mark Clements, Gregory P. Forlenza, Anna Neyman, Lisa Norlander, Marcus Schamberger, Jennifer L. Sherr, Ryan Bailey, Roy W. Beck, Craig Kollman, Shannon Beasley, Erin Cobry, Linda A. DiMeglio, Emily Paprocki, Michelle Van Name, Antoinette Moran, for the CLVer Study Group

**Affiliations:** a University of California, San Francisco, San Francisco, CA, USA; b Stanford University, Palo Alto, CA, USA; c Jaeb Center for Health Research, Tampa, FL, USA; d Children’s Mercy Hospital-Kansas City, Kansas City, MO, USA; e Barbara Davis Center, University of Colorado Anschutz Medical Campus, Denver, CO, USA; f Indiana University School of Medicine, Indianapolis, IN, USA; g Yale School of Medicine, New Haven, CT, USA; h University of Minnesota, Minneapolis, MN, USA

**Keywords:** Type 1 diabetes, Verapamil, Children, Newly diagnosed, Safety

## Abstract

**Objectives:**

To report the safety and side effects associated with taking verapamil for beta-cell preservation in children with newly-diagnosed T1D.

**Research Design and Methods:**

Eighty-eight participants aged 8.5 to 17.9 years weighing ≥ 30 kg were randomly assigned to verapamil (N = 47) or placebo (N = 41) within 31 days of T1D diagnosis and followed for 12 months from diagnosis, main CLVer study. Drug dosing was weight-based with incremental increases to full dosage. Side effect monitoring included serial measurements of pulse, blood pressure, liver enzymes, and electrocardiograms (ECGs). At study end, participants were enrolled in an observational extension study (CLVerEx), which is ongoing. No study drug is provided during the extension, but participants may use verapamil if prescribed by their diabetes care team.

**Results:**

Overall rates of adverse events were low and comparable between verapamil and placebo groups. There was no difference in the frequency of liver function abnormalities. Three CLVer participants reduced or discontinued medication due to asymptomatic ECG changes. One CLVerEx participant (18 years old), treated with placebo during CLVer, who had not had a monitoring ECG, experienced complete AV block with a severe hypotensive episode 6 weeks after reaching his maximum verapamil dose following an inadvertent double dose on the day of the event.

**Conclusions:**

The use of verapamil in youth newly-diagnosed with T1D appears generally safe and well tolerated with appropriate monitoring. We strongly recommend monitoring for potential side effects including an ECG at screening and an additional ECG once full dosage is reached.

ClinicalTrials.gov number: NCT04233034.

## Introduction

Type 1 diabetes (T1D) is an autoimmune condition leading to the gradual loss of pancreatic beta cells, which causes lifetime exogenous insulin dependency and can be associated with long-term micro and macrovascular complications. Retention of islet cell function in people with T1D has been associated with lower hemoglobin A1c (HbA1c) levels, lower risk of hypoglycemia, and reductions in complications [1]. Although the use of technology (continuous glucose monitors and automated insulin delivery systems) improves glycemic outcomes, additional treatments that retain endogenous insulin production would be beneficial. However, most treatments targeting autoimmunity have the potential for significant adverse effects.

Research has shown that thioredoxin-interacting protein (TXNIP) is essential for glucotoxicity-induced beta-cell death in mice and is inhibited by calcium channel blockers such as verapamil [2]. In a randomized trial of 88 children 8.5–17.9 years old with newly-diagnosed stage 3 T1D (CLVer study—Hybrid **C**losed **L**oop Therapy and **Ver**apamil for Beta Cell Preservation in New Onset Type 1 Diabetes), oral verapamil, initiated within 31 days of diagnosis (median 24 days), reduced beta cell decline. Twelve months from diagnosis, mixed-meal tolerance test (MMTT)-stimulated C-peptide levels were 30 % higher in those treated with verapamil compared with placebo [3]. These results were similar to a smaller adult pilot study [4].

Our placebo-controlled trial provided the opportunity to assess the frequency of verapamil side effects during one year of therapy, providing valuable information in developing guidance for initiating and monitoring verapamil therapy in the new-onset pediatric T1D population. Participants in the main study could also participate in an observational extension phase of the study (CLVerEx) with quarterly contacts to obtain continuous glucose monitoring (CGM) and insulin dosage data, and a yearly mixed meal tolerance test for c-peptide levels. Use and management of verapamil in the extension phase was the decision of the patient and their clinical care providers, allowing for assessment of “real-world” experience with implementing verapamil therapy.

## Methods

Study methods for the randomized trial have been published [3], [5] and the protocol is available [3]. Relevant eligibility criteria included age 7–<18 years, diagnosis of antibody-positive T1D within 31 days of randomization, and weight ≥30 kg. Exclusion criteria included baseline hypotension, electrocardiogram (ECG) abnormalities, eGFR < 90, or aspartate transaminase (AST) or alanine transaminase (ALT) greater than 1.5 times the upper limit of normal. One participant in the placebo group dropped from the trial before starting the study drug and was not included in this analysis.

The extended-release formulation of verapamil (ER) was used to allow for once daily dosing with food. The lower weight limit for trial eligibility was related to verapamil dosing constraints. Currently, the lowest dose of the verapamil ER formulation is a 120 mg tablet that can be split to make half tablets (60 mg). Drug dose (verapamil or placebo) was weight-dependent, starting at 60 or 120 mg/day, escalating in 60 mg increments every 2–4 weeks, to a weight-dependent maximum of up to 360 mg/day (usual adult dosage) (Table 1). The final dosage was 4.0–7.2 mg/kg/day.

**Table 1:** Verapamil dose escalation by weight.

Weight (kg)	Initial Dose (mg/day)	Dose escalation	Dose escalation	Dose escalation	Max Dose	Max Dose mg/kg/day
30–34	60 X 4 wks	120 mg			120	4
35–49	60 X 2 wks	120 X 2 wks	180 X 2 wks	240 mg	240	6.9
≥50	120 X 2 wks	180 X 2 wks	240 X 2 wks	300 X 2 wks	360	7.2

### Monitoring for adverse events in CLVer

At screening, 6, 26, and 52-week visits, pulse, blood pressure, creatinine, AST and ALT were measured; and an ECG was obtained. Participants taking study drug (verapamil or placebo) were monitored for constipation (the most commonly reported side effect of verapamil), severe hypoglycemic episodes, hypotension, decreased heart rate, changes in ECG including abnormalities in QTc and PR intervals, changes in liver function (AST and ALT), and reports of dizziness, nausea, or headaches.

The dose of the study drug could be reduced/discontinued at the investigator's discretion if side effects developed or if an abnormality was found on clinical exam, ECG, or blood testing.

The dose also could be adjusted if the participant’s weight changed during the study. Participants had remote safety visits one week after initiation of the study drug and one week after each study drug dose increase, with home measurements of blood pressure and pulse using an FDA approved fully automatic blood pressure monitor with both adult and child cuff sizes (Contec 08A). ALT and AST were measured locally. An ECG was performed at screening, 6, 26, and 52 weeks. Adverse events were recorded using electronic case report forms that were reviewed by the medical monitor in addition to the investigator. Serious Adverse Events (SAE) were classified as death, a life-threatening event, an event that required inpatient hospitalization or resulted in significant disability. A Data and Safety Monitoring Board reviewed compiled safety data at periodic intervals.

### Monitoring for adverse events in CLVerEx

CLVerEx, the ongoing extension study of CLVer, is strictly observational with no study drug provided by the research team. During CLVerEx, use of verapamil is allowed but is the decision of the patient and their clinical team without oversight by research study staff. At the quarterly contacts the participant’s CGM is uploaded, the total daily insulin dose is recorded, and they are asked if there have been any adverse events or changes in medications.

## Results

In CLVer, there were 134 AEs reported for 39 (83 %) of the 47 participants in the verapamil group and 91 AEs reported for 30 (75 %) of the 40 participants in the placebo group. Four participants in the verapamil group and 4 in the placebo group experienced SAEs, none of which were considered to be related to the study drug. In the verapamil group, one participant had a severe hypoglycemic event, and 3 had hospitalizations for suicidal ideation or depression. In the placebo group, the 5 events (one participant had 2 events) were one severe hypoglycemia and hospitalizations for diabetic ketoacidosis, suicidal ideation, exacerbation of asthma, and dehydration with ketosis.

Three of 47 (6.4 %) participants in the verapamil group developed asymptomatic ECG findings of a first degree atrioventricular (AV) block with prolonged PR intervals >200 ms (n = 2) or second degree AV block type 1 (Wenckebach) compared with none of 40 in the placebo group (Table 2). These changes were dose-related and in two participants resolved with a decrease in the verapamil dose. Verapamil was discontinued in the third participant without trying a dose reduction due to investigator discretion.

**Table 2:** Time course and verapamil dosing for participants developing AV block.

Case 1: 11-year-old, 31 kg at randomization						
**Weeks since verapamil started**		Baseline	6	24*	27	
**Verapamil (mg) [mg/kg]**		0	120 [3.4]	240 [6.2]	0	
**PR interval on ECG (ms)**		136	124	340	130	
**ECG report**		Normal	Normal	1st degree heart block	Normal	
Blood Pressure (mmHG) Systolic/Diastolic**		99/54	116/57	107/59	107/59	
Heart Rate (bpm)**		99	102	79	79	

**Case 2: 17-year-old, 96 kg at randomization**						
**Weeks since verapamil started**	Baseline	5	22	22 + 1 day	26	49
**Verapamil (mg) [mg/kg]**	0	240 [2.5]	360 [3.9]	0	0	360 [3.6]
**PR interval on ECG**	155	151	N/A	154	150	269
**ECG report**	Normal	Normal	2nd degree AV block, type 1 (Wenckebach)	Normal	Normal	Prolonged PR
Blood Pressure (mmHG) Systolic/Diastolic**	108/58	108/58	106/59	106/59	106/59	107/57
Heart Rate (bpm)**	64	64	78	78	78	64

**Case 3: 13-year-old, 46 kg at randomization**						
**Weeks since verapamil started**		Baseline	6†	10	22	
**Verapamil (mg) [mg/kg]**		0	180 [3.5]	120 [2.3]	120 [2.2]	
**PR Interval**		178	220	156	164	
**ALT (10**–**35)**		43	75	53	20	
**AST (<44)**		37	39	45	24	
**ECG report**		Normal	1st degree AV block	Normal	Normal	
Blood Pressure (mmHG) Systolic/Diastolic**		95/52	119/58	109/69	99/50	
Heart Rate (bpm)**		69	68	79	77	

**Case 4: 18-year-old, 63 kg, started verapamil in extension study after receiving placebo in RCT with verapamil overdose and acute cardiotoxicity**						
**Timeline**		Baseline at study enrollment	6 weeks post max dose	12 h post admit	36 hrs post admit	72 hrs post admit
**Verapamil (mg) [mg/kg]**		0	360[5.8]***	0	0	0
**PR interval**		145	0	147	131	125
**ECG report**		L anterior fascicular block. Axis deviation suggestive of endocardial cushion defect	Junctional rhythm RBBB and LPFB nonspecific st and t wave changes	Sinus bradycardia left axis deviation nonspecific t wave changes	Sinus rhythm left anterior fascicular block nonspecific t wave changes	Sinus rhythm early transition nonspecific t wave changes
Blood Pressure (mmHG) Systolic/Diastolic**		121/74	70/30	110/51	120/66	133/76
**Heart Rate**		89	43	47	55	86
**Treatment**			Atropine Norepinephrine Dopamine	Dopamine	Dopamine	

*Decision made by investigator to stop verapamil (masked decision).

†ALT elevated and PR prolonged, so dose decreased to 120 mg for duration of study.

**Most recent measurement shown.

***This dose was unintentionally doubled on day of admission

Elevated ALT occurred in 2 participants in each group. One of the verapamil participants with an elevated ALT was also one of the 3 participants with ECG changes (Table 2, Case 3); this participant’s ALT level normalized after a reduction in the verapamil dose. The other participant using verapamil had a transient elevation of ALT at 6 weeks which resolved without a change in dose. Of the 2 placebo cases, one had a transient elevation in ALT at 47 weeks and the other had persistent mild elevation of ALT over four months and study medication (placebo) was stopped.

Symptomatic hypotension was reported as an adverse event for a 10-year-old participant in the verapamil group at 6 weeks. His dose was held and then restarted at 6 months; however, blood pressure readings were again low, so medication was stopped. Bradycardia was reported for one participant in the placebo group. The frequency of other non-serious side effects (given as n/% participants on verapamil; n/% on placebo) included constipation (3/6 %;1/3%), headache (4/9 %;7/18 %), nausea (5/11 %;0), hypotension (1/2 %;0), pedal edema (1/2 %;0), and paresthesia (feet numbness) (1/2 %;0). Gingival hyperplasia has been described with verapamil use but was not seen in this study. Constipation was managed with usual therapy and did not require changes in medication doses.

In total during CLVer, the study drug was discontinued in four participants in the verapamil group, for ECG changes, pain/tingling in the feet, suicide ideation (per investigator discretion), and participant choosing to discontinue the drug. Three participants in the placebo group had a dose reduction without discontinuation (ALT elevation, headache, and headache with near-syncope).

Mean blood pressure and pulse readings obtained one week after each dose increase showed no difference between the verapamil and placebo groups. Growth percentiles for the verapamil and placebo groups at study visits tracked as expected for both groups.

In CLVerEx, 5 participants have thus far been prescribed verapamil. There was one severe adverse event six weeks after an 18 year old male who had been treated with placebo during CLVer reached his maximum dose of 360 mg/d of verapamil (5.8 mg/kg/d). He accidentally took a double dose, one in the morning, and one in the afternoon. In the early evening he became nauseated, dizzy, weak, and was unable to stand. In the emergency department his heart rate was 44 bpm, blood pressure 84/40 mmHg, and his ECG showed a junctional rhythm. Lactic Acid was > 6 mmol/l, CO2 = 20 mmol/L; Glucose = 338 mg/dl, Creatinine 1.9 = mg/dl, BUN = 21 mg/dl; AST = 18 IU/L, ALT = 15 IU/L, GFR = 51.7 ml/min. Drug screen was negative. He was admitted to a cardiac intensive care unit and received intravenous atropine, dopamine, norepinephrine and calcium. Within 3 h his lactic acid was normal, and serum creatinine was normal in 8 h. Within 12 h he had a normal PR interval, and within 48 h he was off all vasopressor support.

## Discussion

Verapamil has been used in children off-label to treat supraventricular tachycardia [6], hypertrophic cardiomyopathy [7], and hypertension [8]. Our data demonstrate that oral verapamil is generally safe and well-tolerated by youth with newly diagnosed T1D with appropriate monitoring. Since this was the first use of verapamil in this population, we were cautious about drug escalation to full dosage and in monitoring for side effects. Blood pressure and pulse measurements one week after each dosage change and liver enzyme results were similar in the verapamil and placebo groups.

Serial ECG tracings in CLVer identified 3 cases with asymptomatic changes. Verapamil is known to have an effect on the AV node causing sinus bradycardia, PR prolongation (1st degree AV block) and 2nd AV heart block Type 1 (“Wenckebach”). These low-grade heart blocks were observed at doses of 3.5, 3.9 and 6.2 mg/kg/d, doses well-tolerated by other participants. The potential susceptibility of these three individuals might be explained by the well-known interindividual variability in verapamil pharmacokinetics [9] or by interindividual variation in verapamil effects on the AV node. It should also be noted that asymptomatic 1st degree AV block is usually considered a benign finding and can be seen incidentally in 11 %–12 % of healthy children and teenagers and in 23 % of teenage athletes [10], [11]. However, since this represented a change from baseline, verapamil doses were lowered in two and discontinued in one participant in an abundance of caution. The half-life of verapamil ER is between 5 and 10 h [12] so holding one dose can be sufficient to resolve an AV block as in case 2.

In CLVerEx, one participant who had originally been on placebo and had subsequently started verapamil experienced a complete (3rd degree) AV block with severe hypotension requiring ICU admission. He had not had an ECG obtained after reaching his maximum dose of verapamil so it is unknown whether he had a prolonged PR interval or other lower grade heart block preceding this event. He recovered completely without sequela. The event occurred after an unintentional double dose of verapamil on the day of the event despite use of a weekly pill organizer box. No additional contributing factors that could have led to the verapamil toxicity were identified in this patient. In the literature most cases of verapamil overdose associated with a complete heart block and severe hypotension are the result of ingestion of well over 1,000 mg of verapamil [13], [14]. There is one case of verapamil cardiotoxicity leading to death with a dose estimated to be 720 mg to 840 mg. However, that patient also had mild, chronic renal failure [15]. In settings outside of T1D, asymptomatic first-degree AV block with PR-interval prolongation can be seen during the early titration phase of verapamil therapy, but higher degrees of AV block are infrequently (0.8 %) observed [16].

We strongly recommend that anyone starting verapamil have a baseline ECG and another ECG within two weeks of reaching their maximal dose. The ECG at maximal dose and a virtual or in person visit to review these results should be scheduled at the time of the verapamil initiation visit to make sure they are not missed. R enantiomer form of verapamil has a higher cardiovascular safety margin but has not been tested in T1D [17].

Verapamil administered concomitantly with oral antihypertensives may have an additive effect on lowering the blood pressure and thus should be used cautiously in this situation [16]. Verapamil is metabolized through the cytochrome P450 (CYP 3A4) pathway similar to statins, protease inhibitors, antifungals, phenobarbital, allopurinol, erythromycin, aspirin, oral contraceptives and other medications [8]. Therefore, drug interactions should be assessed. Verapamil bioavailability is increased by 30 % with grapefruit juice and 10 % with orange juice [18], and verapamil extends the duration of alcohol intoxication [19], [20].

Verapamil can cause elevations in transaminases which are usually transient and may resolve even with continued therapy [16]. Rare anecdotal reports in adults have described hepatic hypersensitivity reactions which resolved two weeks after drug discontinuation [16], [21], [22]. Verapamil should not be restarted in the case of a hypersensitivity reaction.

If health care providers consider initiating extended-release verapamil therapy off-label as a beta cell preservation agent we would suggest, based on this study and our review of the literature, the following:

1) Obtain an ECG before starting verapamil, 2 weeks after achieving the maximum dose, at 6 months, and yearly thereafter. If the PR interval is prolonged, the verapamil dose should be decreased, and the ECG reassessed.
2) Use a weekly pill organizer. If a dose is missed, it should be skipped, and not given as a double dose the following day.
3) Weight-based dose increments by 60 mg for individuals weighing >30 kg, with a minimum dose of 60 mg/day (unless a lower dose extended-release formulation becomes available) up to a maximum dose of 360 mg/day (Table 1).
4) Obtain creatinine AST and ALT and creatinine before starting verapamil and repeat if there are concerns about liver or renal disease.
5) Blood pressure measurements at the time of ECG testing. Consider more frequent or home monitoring in patients with baseline blood pressure in the lower normal quartile for age.
6) When starting new medications, assess their interaction with verapamil.
7) Provide information to the family on
  a. potential side effects seen with verapamil
  b. potential interactions with other drugs, grapefruit juice and alcohol.
8) Taking the drug with food may decrease GI symptoms.

The main limitation of this study is the sample size which may be too small to detect rare side effects.

The dose in this trial was chosen according to a previous study testing verapamil in adults with T1D which demonstrated tolerability and effectiveness, proportionately modified for the smaller size of children. A study to determine the best dose to maximize effect and minimize side effects has not been conducted. Larger future studies are needed to assess whether there is any benefit to starting verapamil sooner in pre-clinical stages of T1D, or later provided there is still residual C-peptide. Additional work is also needed to provide more data on verapamil dosing (including the rate of dose escalation), length of beta cell effect, potential rarer side effects, long-term safety and tolerability, and dosing for children weighing less than 30 kg.

## Data sharing statement

Data will be made available on a publicly available website (www.jaeb.org) at a later date.

## Author’s Contribution

**All of the authors participated in the conduct of the study.** LE and BB wrote and edited the manuscript. RB wrote the manuscript and performed statistical analyses. CB, MC, GPF, AN, LN, MS, JLS, RWB, CK, SH, EC, LAD, EP, MVN and AM reviewed and edited the manuscript.

## Funding

Study funding was provided by JDRF (formerly the Juvenile Diabetes Research Foundation). Medtronic, Tandem Diabetes Care, and Dexcom provided the devices used in the trial.

## CRediT authorship contribution statement

**Laya Ekhlaspour:** Writing – original draft, Investigation, Conceptualization. **Bruce Buckingham:** Writing – original draft, Investigation. **Colleen Bauza:** Writing – review & editing, Project administration, Methodology, Funding acquisition, Formal analysis, Data curation. **Mark Clements:** Writing – review & editing, Investigation. **Gregory P. Forlenza:** Writing – review & editing, Investigation. **Anna Neyman:** Writing – review & editing, Investigation. **Lisa Norlander:** Writing – review & editing, Investigation. **Marcus Schamberger:** Writing – review & editing, Investigation. **Jennifer L. Sherr:** Writing – original draft, Investigation. **Ryan Bailey:** Writing – original draft, Formal analysis. **Roy W. Beck:** Writing – review & editing, Supervision. **Craig Kollman:** Writing – review & editing, Supervision. **Shannon Beasley:** Writing – review & editing, Investigation. **Erin Cobry:** Writing – review & editing, Investigation. **Linda A. DiMeglio:** Writing – review & editing, Investigation. **Emily Paprocki:** Writing – review & editing, Investigation. **Michelle Van Name:** Writing – review & editing, Investigation. **Antoinette Moran:** Writing – review & editing, Investigation.

## Declaration of competing interest

The authors declare the following financial interests/personal relationships which may be considered as potential competing interests: Dr. Ekhlaspour receives salary support from NIH and research support from JDRF. She reports receiving consulting fees from Medtronic, Tandem Diabetes Care and Ypsomed; receiving speaking fees from Insulet; and receiving research support from Medtronic, MannKind and Abbot through her institution. Her prior institution has received research support from Medtronic, Tandem Diabetes Care, Insulet, Dexcom and Beta Bionics. Dr. Buckingham reports receiving grants, personal fees, and/or nonfinancial support from Medtronic, Tandem Diabetes Care, Insulet, NovoNordisk, and Lilly and reports his institution has received research funding from Medtronic, Tandem Diabetes Care, Beta Bionics, and Insulet. Dr. Bauza reports no disclosures. Dr. Clements reports receiving personal fees from Glooko Inc and nonfinancial support from Dexcom and Abbott Diabetes Care. Dr. Forlenza reports serving as a consultant, speaker or advisory board member for Medtronic, Dexcom, Abbott, Tandem Diabetes Care, Insulet, Lilly, and Beta Bionics and reports his institution has received funding on his behalf for research grants from Medtronic, Dexcom, Abbott, Tandem Diabetes Care, Insulet, Lilly, and Beta Bionics. Dr. Neyman reports no disclosures. Dr. Norlander reports no disclosures. Dr. Schamberger reports no disclosures. Dr. Sherr reported receiving speaking honoraria from Lilly, Insulet, Medtronic, and Zealand Pharma; serving on advisory boards for Bigfoot Biomedical, Cecelia Health, Insulet, Medtronic Diabetes, JDRF (formally the Juvenile Diabetes Research Foundation) T1D Fund, StartUp Health T1D Moonshot, and Vertex Pharmaceuticals; receiving consultant fees from Insulet and Medtronic; and reported that her institution has received research grant support from Medtronic and Insulet. Mr. Bailey reports no disclosures. Dr. Beck reports his institution has received funding on his behalf as follows: grant funding and study supplies from Tandem Diabetes Care, Beta Bionics, and Dexcom; grant funding from Bigfoot Biomedical; study supplies from Medtronic, Ascencia, and Roche; consulting fees and study supplies from Lilly and NovoNordisk; and consulting fees from Insulet and Zucara Therapeutics. Dr. Kollman reports receiving grants from Dexcom and Tandem Diabetes Care. Ms. Beasley reports no disclosures. Dr. Cobry reports no disclosures. Dr. DiMeglio reports receiving consulting or advisory fees from Abata Therapeutics, MannKind, Provention Bio, and Zealand Pharma and study supplies from Dexcom. Dr. Paprocki reports no disclosures. Dr. Van Name reports receiving research support from ProventionBio. Dr. Moran reports serving on advisory boards from Dompé Farmaceutici SpA, ProventionBio, and Abata Therapeutics; serving on a data and safety monitoring board for NovoNordisk; and reported that her institution has received grant funding on her behalf from Abbott Diabetes, ProventionBio, Intrexon (now Precigen), and Caladrius Biosciences and study supplies from NovoNordisk, Medtronic, and Abbott Diabetes.
